# Determinants of Preventive Behaviors in Response to the COVID-19 Pandemic in France: Comparing the Sociocultural, Psychosocial, and Social Cognitive Explanations

**DOI:** 10.3389/fpsyg.2020.584500

**Published:** 2020-11-30

**Authors:** Jocelyn Raude, Jean-Michel Lecrique, Linda Lasbeur, Christophe Leon, Romain Guignard, Enguerrand du Roscoät, Pierre Arwidson

**Affiliations:** ^1^EHESP School of Public Health, Rennes, France; ^2^Unite des Virus Emergents (UVE: Aix-Marseille Univ – IRD 190 – Inserm 1207 – IHU Mediterranee Infection), Marseille, France; ^3^Santé publique France, Saint Maurice, France; ^4^Laboratoire Parisien de Psychologie Sociale (LAPPS), EA 4386, Université Paris Ouest Nanterre-La Défense, Nanterre, France

**Keywords:** preventive behavior, social cognition, COVID-19, risk perception (RP), social norm, adherence - compliance - persistance, social determinansts of health, psychosocial factors

## Abstract

In absence of effective pharmaceutical treatments, the individual's compliance with a series of behavioral recommendations provided by the public health authorities play a critical role in the control and prevention of SARS-CoV2 infection. However, we still do not know much about the rate and determinants of adoption of the recommended health behaviors. This paper examines the compliance with the main behavioral recommendations, and compares sociocultural, psychosocial, and social cognitive explanations for its variation in the French population. Based on the current literature, these 3 categories of factors were identified as potential determinants of individual differences in the health preventive behaviors. The data used for these analyses are drawn from 2 cross-sectional studies (*N* = 2,000 in survey 1 and 2,003 in survey 2) conducted after the lockdown and before the peak of the COVID-19 epidemic in France. The participants were drawn from a larger internet consumer panel where recruitment was stratified to generate a socio-demographically representative sample of the French adult population. Overall, the results show a very high rate of compliance with the behavioral recommendations among the participants. A hierarchical regression analysis was then performed to assess the potential explanatory power of these approaches in complying with these recommendations by successively entering sociocultural factors, psychosocial factors, social cognitive factors in the model. Only the inclusion of the cognitive variables substantially increased the explained variance of the self-reported adoption of preventive behaviors (*R*^2^ change = 23% in survey 1 and 2), providing better support for the social cognitive than the sociocultural and psychosocial explanations.

## Introduction

With the emergence and rapid spread of the SARS-CoV-2 through the world has raised the Specter of a novel and potentially catastrophic pandemic of a highly contagious and severe respiratory disease, with social, economic, and health consequences comparable to those of the well-known “Spanish flu” pandemic of 1917–18. In the absence of known effective pharmaceutical products to treat patients, the spread of the virus has greatly affected public health systems and healthcare services across the planet. In Europe, a variety of public health strategies have been adopted by governments and policy-makers to prevent the transmission of COVID-19 and control the epidemic at the national and regional level (Hunter et al., 2020). However, due to the relative authorities' unpreparedness to this unexpected and unprecedented situation, most European countries were not able to rely on the sole implementation of a strategy of prevention, essentially based on a high level of diagnosis capacities and isolation of infected patients. Instead, most governments implemented a population strategy based on the administration of non-pharmaceutical interventions designed to control the spread of the disease through social and health behavior change (West et al., 2020). In practice, these public health interventions ranged from the non-coercive promotion of social distancing and improved hygiene measures (in Sweden) to government-imposed lockdowns (in France, Spain, or Italy).

From an epidemiological perspective, human behaviors play a fundamental role in the propagation of many pathogens by either amplifying or attenuating their transmission through person-to-person contact (Ferguson, 2007; Bauch et al., 2013). There is now substantial evidence showing that large-scale adoption of preventive behaviors by individuals and communities, through improved personal hygiene or social distancing measures, is generally effective in lessening the impact of epidemic of acute respiratory diseases by reducing and slowing down the transmission (Cowling et al., 2020). Therefore, in France like in most developed countries, the course of the COVID-19 epidemic depends to a large extent on the manner populations comply with the regulations and adhere to the behavioral recommendations provided by the public health authorities. However, we still do not know much about how people respond to the COVID-19 epidemic, as well as the causes and motives of the engagement in the health protective behaviors recommended by the national and regional governments (Van Bavel et al., 2020). To date, only a few exploratory studies investigated the determinants of health behavior compliance in the general population. Moreover, these studies led to contradictory results. For instance, some investigators found that the perceived risk of infection was strongly associated with increased engagement in health behaviors (Berg and Lin, 2020; Bruine de Bruin and Bennett, 2020), while others showed that this construct was not an important predictor after controlling for sociodemographic variables (Clark et al., 2020). In order to develop more successful and suitable interventions for all or specific subpopulations, it is important to improve the understanding of the psychological and social factors that affect the compliance with and adherence to the regulations and recommendations.

## Theoritical Background

Psychological explanations and models of adherence to various behavioral recommendations aiming to preserve health and prevent diseases have significantly changed in the last decades. Early research has led to the development of a range of social cognitive models, which focus on the role of health-related beliefs and expectations and their effects on motivation to take actions, as key determinants of subsequent individual's adherence to behavioral recommendations. Many of these psychological models—such as the Rosenstock's Health Beliefs Model (Champion and Skinner, 2008), the Roger's Protection Motivation Theory (Conner and Norman, 2005), or the Fishbein and Ajzen's Theory of Planned Behaviors (Armitage and Christian, 2003)—can be related to more general theories of human behavior based on expectancy and value (for a review, see Armitage and Conner, 2000). This theoretical framework assumes that motivation to engage oneself in a specific behavior or action is determined by the combination of two factors: (1) expectancy, which refers to how probable one think that a given outcome is likely to occur by taking the action, and (2) value, which refers to how much one values the anticipated outcome(s). Applied to health issues, expectancy-value models generally highlight the importance of a broad range of beliefs that individuals have about health threats and the measures to prevent them, as well as their own capability to execute the recommendations which are provided to them. Today, expectancy-value theories such as HBM or TPB are undoubtedly the most common used models in psychological literature to explain the adoption of health protective behaviors (Conner and Norman, 2005). Recent reviews of literature also have shown that the explanatory variables drawn from these models are relatively valuable to predict how people react to emerging infectious diseases, such as SARS or H1N1 pandemic influenza (Smith, 2006; Leppin and Aro, 2009; Bish and Michie, 2010; Taylor, 2019).

However, while these leading models derived from the early stage of health behavior research have identified a range of cognitive and affective factors that underpins the adherence (or non-adherence) to behavioral recommendations at the individual level, they are not helpful to explain why health behaviors and their determinants tend to vary between subgroups of populations. As suggested by Wong and Jensen (2020), there is with regard COVID-19 prevention a need for “further investigation into other social and cultural factors that may have stronger influence over individual belief in the need of personal actions to control the risks.” For instance, gender differences have been consistently found in the way individuals perceive and respond to health-related risks across a variety of countries and domains (environmental, technological, societal), regardless of age, level of education, or even professional status (Finucane et al., 2000; Raude et al., 2005; Kahan et al., 2007). Notably, a recent meta-analysis shows that female respondents were about 50% more likely than their male counterparts to engage in health protective behaviors in response to epidemic and pandemic respiratory infectious diseases (Moran and Del Valle, 2016). Interestingly, this gender gap in the behavioral response to health threat was also found for the COVID-19 pandemic across countries (Clark et al., 2020).

In the same vein, a series of socioeconomic disparities in physical and mental health have long been observed, even within the richest and most developed nations of the world. Numerous epidemiological studies have shown that these social inequalities in health were due to a large extent by the existence of socially differentiated patterns of lifestyles and habits in populations, such as tobacco smoking, alcohol consumption, physical activity, or food habits (Stringhini et al., 2010; Nandi et al., 2014). In other words, socioeconomic differences in the practice of a number of health behaviors have been found as one of the most important pathways through which social conditions affect individual health, not only in relation to non-communicable diseases but also through infectious disease (Cohen et al., 2007). To further account for the higher prevalence of unhealthy habits and actions as well as of non-adherence to common health recommendations, among the most disadvantaged groups, several psychosocial explanations have been offered in the literature. Research conducted over the past 30 years have noticeably led to identify 3 factors that may play a major role in explaining socioeconomic differences in health status: trust in institution, social support, and anxiety. For instance, some studies in health psychology have provided empirical evidence that differences in anxiety caused by more stressful life and labor conditions among disadvantaged groups contribute to social disparities in the engagement in health protective behavior (see Schneiderman et al., 2001, for a review). Indeed, anxiety and poverty seem to influence the importance that people give to health preservation or improvement because it is already difficult for them to deal with existing demands (Evans and Kim, 2013). Other studies have shown that the effect of trust in institutions on public acceptance of some health-related innovations or interventions is mediated to a large extent by cognitive variables such as the perceived risk and benefits (Visschers and Siegrist, 2008; Bronfman and Vázquez, 2011; Plohl and Musil, 2020).

Overall, these empirical results suggest that efforts to promote health protective behaviors should be based on an understanding of multidimensional and complex interplay among various cognitive, psychosocial and sociocultural factors, rather than on analyses that focus solely on psychological or social factors. Thus, the principal aim of the present study is to examine the contribution of the 3 categories of explanatory variables presented above—sociocultural (gender, age, and socioeconomic status), psychosocial (trust, anxiety, and social support), and cognitive (beliefs and expectations)—to the individual variation in the compliance with regulations and adherence to behavioral recommendations provided by the public health authorities to tackle the COVID-19 epidemic. The secondary aim is to assess the ability of psychosocial and cognitive variables to mediate the effect of sociocultural and demographic variables on health protective behaviors. Based on the above-cited literature, it was expected that both social cognitive factors and psychosocial factors account for the relationship between the sociocultural and demographic characteristics of the participants and their degree of compliance with the public health recommendations.

## Analytic Strategy

In health psychology, two main analytic strategies can be identified: the summary approach and the splitting approach. The former consists of testing health behavior theories—defined as a specific and coherent system of causal relations among constructs—against one another. The summary approach has been increasingly advocated by some authors to improve cumulative knowledge in health behavior research (Noar and Zimmerman, 2005; Weinstein and Rothman, 2005). However, the comparative testing of whole theories has raised a number of epistemological criticisms. As noted by Weinstein and Rothman (2005, p. 296): “theories are not static entities to be used as initially proposed, but rather are dynamic entities that should evolve over time. Theory improvement is a cyclical process that involves the specification of relations between factors, the testing of those relations, the re-specification or rejection of initially hypothesized principles and the testing of the new relations.” This led some authors to promote an alternative approach, which consists of testing competitive hypotheses drawn from diverse models. For instance, Brewer and Gilkey (2012) have convincingly argued that it is possible to disassemble health behaviors theories into more elementary components so that specific aspects from different theoretical frameworks can be compared. In our opinion, this latter approach is more promising for advancing the knowledge about the determinants of health protective behaviors in response to emerging infectious diseases than the summary approach.

## Methods

### Participants and Procedures

Our data was collected through online surveys conducted among large samples of adults residing in France (https://www.bva-group.com/en/about-us/). The samples were recruited among respondents aged 18–90 years old who agree to participate regularly to surveys of customer attitudes and experiences in exchange for financial compensation. The participants in each of these surveys were enrolled on the basis of a stratified sampling method to reflect the distribution of the French general population regarding sex, age, occupation, community size, and region based on the 2016 general census of the population of the National Institute of Statistics and Economic Studies (INSEE). The total samples consisted of 2,000 participants in Survey 1 and 2,003 in Survey 2, of whom 16.9 and 15.8%, respectively, reported to have personally suffered from signs or symptoms indicating a possible SARS-CoV2 infection. Within the samples, more than half of these participants were women (52% in S1 and 52.4% in S2), and 29/29.8% had a high socioeconomic status, 31/31.4% had a low socioeconomic status, and another 39.7/39.2% were inactive (retired, students and persons engaged in activities in the household) in S1 and S2, respectively. Consistent with previous analyses of online surveys of panelists, the participation rates were significantly higher among people from more socioeconomically advantaged categories. Ages ranged from 18 to 89 years, with a proportion of participants aged 65 years or older of 23.9 and 24.0% in S1 and S2, respectively. No significant differences were observed regarding the sociodemographic and health characteristics of the participants between the samples (*p* > 0.05). For the present study, we analyzed data from the first two surveys of this national study, which were administered just after the implementation of the full lockdown (between 23 and 25 March 2020) then before the peak of the epidemic (between 30 March and 1 April 2020) in France. The research protocol was registered by the EHESP School of Public Health Office for Personal Data Protections and approved by the ethical committee of the University Hospital Institute “Mediterranee Infection” (Marseille, France).

### Measures

#### Compliance With Behavioral Recommendations

The dependent variable for the analyses was the adoption of a range of preventive behaviors recommended by the public health authorities in France to tackle the COVID-19 pandemic. These behavioral changes were used as a proxy variable to capture the compliance with the public health recommendations and guidance about COVID-19. At the time of these surveys, seven health protective behaviors were more specifically recommended to the population to prevent the infection, including “Wash hands often,” “Cover mouth and nose with a tissue or sleeve when coughing or sneezing,” “Use a tissue for each sneeze then throw it in the trash,” “Do not shake hands,” “Stay home as much as possible,” “Avoid close contacts with other people,” “Stay at least 1 m away from other people.” In the surveys, participants were asked whether they practiced each of these behaviors to reduce the risk of COVID-19 infection. The possible response options were “Yes” and “No” in Survey 1, then “Yes, systematically,” “Yes, often,” “Yes, sometimes,” and “No, never” in Survey 2. Given the ceiling effect observed in survey 1 (the majority of values approached the upper limit of the scale), responses obtained in survey 2 from these seven items were dichotomized with the “high compliance” response option (“Yes, systematically”) coded as 1, and the other options (“Yes, often,” “Yes, sometimes,” and “No, never”) combined into a “lower compliance” category coded as 0. To reduce the skewness and increase variance of the dependent variable, we deliberately chose not to combine the positive options (“Yes, systematically,” ‘Yes, often,” and “Yes, sometimes”) into a “Yes” category. Indeed, data collected through the whole surveys showed that about 90% of the participants responded “Yes, systematically,” compared to about 1% who responded “No, never.” Finally, the values for each item were added to generate a cumulative score (range 0–7) that enables to measure participants' compliance with the behavioral recommendation.

#### Cognitive Factors

To assess participant's beliefs and expectations related to the COVID-19 epidemic, we used a wide range of constructs and variables drawn from the leading social cognitive models of health behavior (such as the Health Belief Model, the Protection Motivation Theory, or the Planned Behavior Theory). This includes perceived susceptibility (“How vulnerable do you feel to coronavirus (COVID-19)?”) to and severity (“How severe do you think the coronavirus (COVID-19) is?”) of the coronavirus infection, worry (“How worried are you about getting the coronavirus (COVID-19)?,” perceived behavioral control (“How capable do you think you are to adopt protective behaviors?”). These cognitive factors were assessed with single items based on the format and phrasing of questions commonly used in the health psychology literature (Brewer et al., 2007; Ferrer et al., 2016). For each of them, the participants were asked to rate on an 11-point Likert scale ranging from 0 to 10 in which the meaning of the end-point values was explicitly indicated.

We also included in the analysis four cognitive variables based on multi-items scales, which were perceived barriers (2 items in S1-7 items in S2, e.g. “How difficult do you think it is to adopt improved hygiene measures to prevent the COVID-19 infection?,” Cronbach's alpha = 0.86 and 0.73 in Survey 1 and 2, respectively) and perceived effectiveness of the preventive behaviors recommended by the public health authorities (2 items in S1, 7 items in S2), e.g. “How effective do you think the improved hygiene measures are to prevent the COVID-19 infection,” Cronbach's alpha = 0.74 and 0.82 in Survey 1 and 2, respectively), perceived cause of infection (5 items in S1- 9 items in S2), e.g., “Can coronavirus be transmitted by people without symptoms?”) and subjective norms (4 items, e.g., “Most people who are important to me approve that I have adopted improved hygiene measures to prevent the COVID-19 infection,” Cronbach's alpha = 0.75 and 0.77 in Survey 1 and 2, respectively). In this last case, participants were asked to select one of four response options (strongly disagree, disagree, agree, strongly agree), and responses were summed across items to generate scores on the scale (possible scores: 4–16).

#### Psychosocial Variables

Anxiety was assessed with the seven-item version of the Zigmond and Snaith's Hospital Anxiety and Depression scale (HAD) (possible scores: 0–21), which had a Cronbach's alpha of 0.82 and 0.82 in the survey 1 and 2 of the present study (Zigmond and Snaith, 1983). Social support was measured with a set of items measuring the various dimensions of social support (emotional, instrumental, and informational) drawn from a social and epidemiological cohort study carried out in Paris (Chauvin and Parizot, 2009). As this 3-items scale showed a low internal consistency (Cronbach's alpha <0.60), the items were entered separately in the analysis. Trust in government was also assessed with two questions adapted from the existing literature (van der Weerd et al., 2011): “How much do you trust the authorities to inform you about the Coronavirus (COVID-19)?,” and “How much do you trust the authorities to control the epidemic of Coronavirus (COVID-19)?” (Cronbach's alpha = 0.91 and 0.93 in surveys 1 and 2, respectively). Participants were again asked to rate their response on an 11-point Likert scale ranging from 0 (complete distrust) to 10 (complete trust). The responses were then summed across items to generate scores on a trust in government scale (range 0–20).

#### Sociocultural and Demographic Factors

The questionnaire included a wide range of items aimed at collecting sociocultural and demographic information such as age, gender, level of education, occupational status (student, employed full-time, employed part-time, unemployed at present, or retired), household income, size of household and housing conditions. In addition, participants were asked whether (1) since the start of the epidemic hey had personally suffered from a range of symptoms that can be related to a SARS-CoV2 infection (e.g., “fever,” “dry cough,” or “difficulty breathing”) that could indicate a coronavirus infection (response options: “Yes,” “No,” or “Not sure”) and whether (2) they utilized a range of healthcare service, such as General Practitioners or Emergency services (response options: “Yes” or “No”).

### Data Analyses

Means and standard deviations were calculated for all measures related to the social cognitive and psychosocial variables. To compare differences in scores among participants, according to their sociocultural, health and demographic characteristics, adjusted Wald tests were utilized. To detect and assess relationships between the various variables included in the analysis, Pearson correlations were calculated. To examine the relation between these 3 classes of determinants and compliance with behavioral recommendations, a hierarchical regression analysis was performed. The first step just included the sociocultural and health variables (age, sex, socioeconomic status, and history of COVID-19 infection) to estimate their effect on the participants' degree of adoption of preventive measures. The psychosocial variables (anxiety, social support, and trust in government) were included on the step 2, and the social cognitive variables on step 3. The relative predictive validity of the different types of explanations for compliance with behavioral recommendations (i.e., sociocultural, psychosocial, and social cognitive) was evaluated by examining the percent accounted for each class of determinants (adjusted *R*^2^ values), and the standardized beta (β) associated with each individual variables.

Finally, to further investigate the multiple and complex interactions among the 3 categories of determinants, we performed a series of tests to assess indirect effects in the context of multiple mediation through Structural Equation Modeling (SEM) program. In accordance with the product-of-coefficients strategy described by Preacher and Hayes (2008), which is an extension for multiple mediator models of the classic approach developed by Sobel, we examined whether sociocultural differences in psychosocial and social cognitive variables may explain sociocultural differences in compliance with behavioral recommendations. Because the sociocultural characteristics of the participants are generally not suitable variables for intervention, it is important to determine whether other variable account for the relation between sociodemographic variables and preventive behaviors. The total and specific indirect effects were evaluated by examining the asymptotic critical ratios (*Z*) and Standard Errors (SE). Statistical significance was a priori defined by a *p*-value below 5%. All data was treated and analyzed using STATA (version 15).

## Results

### Compliance With Behavioral Recommendation

Overall, participants self-reported a very high degree of adoption of the main behavioral recommendations provided by the public health authorities. Indeed, the rate of adoption of each of the seven above-mentioned recommendations was systematically higher than 85% in both surveys, ranging from 87% for “Use a tissue for each sneeze then throw it in the trash” in S1 to 99% for “Wash hands often” and “Do not leave home as much as possible” in S2. As presented in Table 1, the mean number of recommended preventive behaviors adopted by the participants was 6.6 (95% CI: 6.6–6.7) in Survey 1 vs. 5.7 (95% CI: 5.6–5.7) in Survey 2. However, these scores cannot be compared as the measurement method of the behavioral variables was slightly modified between the two surveys, with a shift from 2 to 4 response options to reduce the ceiling effect, i.e., the skewness and little variance observed in the compliance with the public health recommendations in the first survey (see *Measures* section). These results imply that the study explored the determinants that divide those participants who vigorously took preventive actions and those who less vigorously took them during that early pandemic period.

**Table 1:** **(A)** Means and 95% Confidence Intervals for Means on the scales (Survey 1, *N* = 2,000); **(B)** Means and 95% Confidence Intervals for Means on the scales (Survey 2, *N* = 2,003).

**Sociocultural and demographic factors**	**Frequencies**	**Adoption of 7 preventive behaviors**	**Anxiety (HAD scale)**	**Trust in institutions**	**Perceived effectiveness of preventive behaviors**	**Perceived barriers of preventive behaviors**	**Subjective norms**	**Perceived behavioral control**	**Worry**	**Perceived severity**	**Perceived susceptibility**	**Perceived cause of infection**
**(A)**												
All participants	2,000	6.64 [6.61–6.68]	7.82 [7.63–8.01]	10.85 [10.61–11.09]	6.84 [6.81–6.87]	1.63 [1.53–1.72]	14.13 [14.05–14.21]	8.61 [8.53–8.68]	7.42 [7.32–7.51]	8.1 [8.02–8.18]	5.79 [5.66–5.91]	8.54 [8.50–8.58]
Gender		*F*_(1,1999)_ = 35.0^***^	*F*_(1,1999)_ = 59.7^***^	*F*_(1,1855)_ = 5.3^*^	*F*_(1,1830)_ = 22.1^***^	*F*_(1,1999)_ = 10.9^***^	*F*_(1,1999)_ = 21.6^***^	*F*_(1,1957)_ = 17.6 ^***^	*F*_(1,1961)_ = 27.1^***^	*F*_(1,1944)_ = 32.5^***^	*F*_(1,1846)_ = 1.29	*F*_(1,1999)_ = 10.4^**^
Male	960	6.53 [6.47–6.59]	7.06 [6.79–7.32]	11.14 [10.79–11.48]	6.76 [6.71–6.82]	1.79 [1.65–1.94]	13.93 [13.81–14.06]	8.45 [8.34–8.55]	7.15 [7.01–7.29]	7.85 [7.73–7.97]	5.71 [5.52–5.90]	8.48 [8.41–8.54]
Female	1,040	6.75 [6.70–6.79]	8.51 [8.25–8.77]	10.6 [10.27–10.90]	6.91 [6.88–6.94]	1.48 [1.36–1.60]	14.32 [14.21–14.42]	8.75 [8.65–8.84]	7.66 [7.53–7.78]	8.33 [8.22–8.44]	5.86 [5.68–6.03]	8.60 [8.56–8.65]
Age group		*F*_(4,1996)_ = 2.5^*^	*F*_(4,1996)_ = 17.5^***^	*F*_(4,1852)_ = 6.2^***^	*F*_(4,1827)_ = 5.9^***^	*F*_(4,1996)_ = 9.6^***^	*F*_(4,1996)_ = 12.2^***^	*F*_(4,1954)_ = 7.9^***^	*F*_(4,1958)_ = 4.6^***^	*F*_(4,1941)_ = 9.8^***^	*F*_(4,1843)_ = 43.0^***^	*F*_(4,1996)_ = 2.31
18–24	196	6.40 [6.22–6.59]	8.90 [8.29–9.51]	11.36 [10.64–12.09]	6.69 [6.56–6.83]	1.95 [1.63–2.27]	13.74 [13.44–14.04]	8.26 [7.98–8.53]	6.91 [6.57–7.26]	7.55 [7.25–7.85]	4.84 [4.42–5.25]	8.33 [8.14–8.51]
25–34	306	6.66 [6.55–6.76]	8.65 [8.15–9.14]	10.40 [9.80–11.00]	6.70 [6.58–6.81]	2.08 [1.82–2.34]	13.77 [13.55–13.98]	8.48 [8.28–8.67]	7.23 [7.00–7.46]	7.85 [7.64–8.07]	4.82 [4.49–5.14]	8.58 [8.48–8.69]
35–49	508	6.67 [6.60–6.73]	8.25 [7.87–8.64]	10.25 [9.78–10.72]	6.86 [6.81–6.91]	1.76 [1.57–1.95]	13.99 [13.83–14.14]	8.48 [8.33–8.62]	7.39 [7.20–7.58]	7.98 [7.81–8.16]	5.43 [5.17–5.69]	8.52 [8.45–8.59]
50–64	512	6.70 [6.65–6.75]	7.48 [7.12–7.83]	10.67 [10.21–11.14]	6.88 [6.84–6.93]	1.43 [1.26–1.61]	14.32 [14.18–14.47]	8.68 [8.55–8.81]	7.66 [7.48–7.84]	8.36 [8.20–8.51]	6.02 [5.78–6.27]	8.60 [8.54–8.67]
65 +	478	6.65 [6.59–6.71]	6.71 [6.37–7.06]	11.73 [11.27–12.20]	6.93 [6.89–6.97]	1.26 [1.09–1.43]	14.5 [14.34–14.65]	8.89 [8.77–9.01]	7.52 [7.33–7.72]	8.34 [8.19–8.49]	6.93 [6.72–7.14]	8.57 [8.50–8.64]
Socioeconomic status		*F*_(2,1998)_ = 2.06	*F*_(2,1998)_ = 16.5^***^	*F*_(2,1854)_ = 5.70^***^	*F*_(2,1829)_ = 3.6^*^	*F*_(2,1998)_ = 3.9^*^	*F*_(2,1998)_ = 5.4^**^	*F*_(2,1956)_ = 4.5^*^	*F*_(2,1960)_ = 1.54	*F*_(2,1943)_ = 3.4^*^	*F*_(2,1845)_ = 0.79	*F*_(2,1998)_ = 13.3^***^
High	962	6.62 [6.58–6.67]	7.27 [7.01–7.54]	11.22 [10.89–11.55]	6.86 [6.82–6.90]	1.50 [1.38–1.62]	14.26 [14.15–14.38]	8.70 [8.60–8.79]	7.34 [7.21–7.48]	8.00 [7.88–8.12]	5.87 [5.69–6.05]	8.64 [8.60–8.69]
Low	805	6.69 [6.63–6.74]	8.23 [7.94–8.52]	10.61 [10.23–10.98]	6.80 [6.75–6.86]	1.71 [1.55–1.87]	14.05 [13.92–14.18]	8.47 [8.35–8.59]	7.52 [7.37–7.67]	8.23 [8.10–8.36]	5.71 [5.52–5.91]	8.47 [8.41–8.53]
Inactive	233	6.57 [6.44–6.71]	8.70 [8.13–9.28]	10.02 [9.27–10.76]	6.91 [6.85–6.96]	1.89 [1.58–2.20]	13.88 [13.64–14.13]	8.67 [8.46–8.89]	7.39 [7.08–7.69]	8.10 [7.85–8.34]	5.68 [5.27–6.10]	8.36 [8.22–8.51]
Covid−19 symptoms		*F*_(1,1999)_ = 1.88	*F*_(1,1999)_ = 36.3 ^***^	*F*_(1,1855)_ = 10.9^***^	(1,1830) = 1.78	*F*_(1,1999)_ = 1.37	*F*_(1,1999)_ = 1.62	*F*_(1,1957)_ = 0.68	*F*_(1,1961)_ = 3.28	*F*_(1,1944)_ = 1.56	*F*_(1,1846)_ = 15.45	*F*_(1,1999)_ = 0.13
No	1,724	6.65 [6.62–6.69]	7.58 [7.38–7.78]	11.01 [10.76–11.26]	6.85 [6.82–6.88]	1.65 [1.55–1.75]	14.16 [14.07–14.24]	8.62 [8.54–8.69]	7.38 [7.28–7.49]	8.08 [7.99–8.17]	5.68 [5.55–5.82]	8.54 [8.50–8.58]
Yes	276	6.58 [6.47–6.68]	9.29 [8.77–9.81]	9.82 [9.16–10.48]	6.78 [6.67–6.88]	1.50 [1.27–1.73]	14.00 [13.77–14.23]	8.53 [8.33–8.73]	7.63 [7.38–7.87]	8.23 [8.01–8.45]	6.41 [6.07–6.74]	8.56 [8.45–8.67]
**(B)**												
All participants	2,003	5.66 [5.58–5.73]	7.31 [7.13–7.50]	11.03 [10.80–11.27]	7.67 [7.59–7.75]	5.23 [5.10–5.37]	14.46 [14.39–14.54]	8.52 [8.45–8.59]	7.39 [7.29–7.48]	8.22 [8.14–8.30]	5.71 [5.57–5.84]	4.56 [4.53–4.59]
Gender		*F*_(1,2002)_ = 61.1^***^	*F*_(1,2002)_ = 45.7^***^	*F*_(1,1930)_ = 4.7^*^	*F*_(1,1910)_ = 13.7^***^	*F*_(1,1965)_ = 46.9^***^	*F*_(1,2002)_ = 28.5^***^	*F*_(1,1972)_ = 27.7^***^	*F*_(1,1983)_ = 12.2^***^	*F*_(1,1983)_ = 13.7^***^	*F*_(1,1863)_ = 1.94	*F*_(1,2002)_ = 1.23
Male	954	5.35 [5.24–5.85]	6.66 [6.40–6.91]	10.76 [10.40–11.11]	7.51 [7.38–7.63]	5.71 [5.53–5.90]	14.26 [14.14–14.37]	8.33 [8.22–8.44]	7.20 [7.05–7.35]	8.06 [7.94–8.19]	5.81 [5.62–6.00]	4.54 [4.49–4.59]
Female	1,049	5.94 [5.47–6.03]	7.91 [7.65–8.14]	11.28 [10.97–11.59]	7.81 [7.71–7.92]	4.80 [4.62–4.99]	14.65 [14.56–14.75]	8.70 [8.62–8.79]	7.55 [7.43–7.68]	8.36 [8.26–8.47]	5.62 [5.43–5.80]	4.58 [4.54–4.62]
Age group		*F*_(4,1999)_ = 7.7^***^	*F*_(4,1999)_ = 7.7^***^	*F*_(4,1927)_ = 6.6^***^	*F*_(4,1907)_ = 15.6^***^	*F*_(4,1962)_ = 0.89	*F*_(4,1999)_ = 12.7^***^	*F*_(4,1969)_ = 9.1^***^	*F*_(4,1980)_ = 11.4^***^	*F*_(4,1980)_ = 10.8^***^	*F*_(4,1860)_ = 50.8^***^	*F*_(4,1999)_ = 2.29
18–24	173	5.13 [4.84–5.42]	7.54 [6.90–8.18]	10.88 [10.15–11.60]	7.39 [7.13–7.65]	5.53 [5.10–5.96]	14.09 [13.84–14.34]	8.51 [8.25–8.78]	6.39 [6.00–6.78]	7.59 [7.30–7.89]	4.05 [3.61–4.50]	4.61 [4.50–4.72]
25–34	294	5.53 [5.33–5.74]	7.94 [7.48–8.41]	10.83 [10.22–10.22]	7.44 [7.23–7.66]	5.23 [4.91–5.55]	14.43 [14.23–14.62]	8.25 [8.06–8.45]	7.16 [6.92–7.41]	8.00 [7.79–8.21]	4.8 [4.46–5.14]	4.63 [4.55–4.71]
35–49	533	5.60 [5.46–5.75]	7.83 [7.47–8.18]	10.51 [10.02–10.02]	7.47 [7.31–7.63]	5.24 [4.99–5.50]	14.25 [14.10–14.39]	8.36 [8.23–8.50]	7.38 [7.19–7.56]	8.17 [8.02–8.33]	5.38 [5.11–5.64]	4.59 [4.53–4.66]
50–64	523	5.77 [5.63–5.90]	6.8 [6.45–7.15]	10.75 [10.29–10.29]	7.67 [7.50–7.85]	5.28 [5.02–5.55]	14.50 [14.36–14.64]	8.56 [8.44–8.69]	7.61 [7.43–7.80]	8.35 [8.20–8.51]	6.10 [5.85–6.35]	4.5 [4.44–4.57]
65 +	480	5.91 [5.78–6.03]	6.79 [6.42–7.17]	12.07 [11.62–11.62]	8.14 [8.01–8.27]	5.05 [4.77–5.34]	14.84 [14.72–14.97]	8.83 [8.71–8.96]	7.73 [7.54–7.91]	8.54 [8.40–9.68]	6.93 [6.70–7.15]	4.53 [4.46–4.59]
Socioeconomic status		*F*_(2,2001)_ = 1.63	*F*_(2,2001)_ = 10.1^***^	*F*_(2,1929)_ = 19.2^***^	*F*_(2,1909)_ = 6.6^**^	*F*_(2,1964)_ = 2.60	*F*_(2,2001)_ = 4.7^**^	*F*_(2,1971)_ = 4.7^**^	*F*_(2,1982)_ = 3.9^*^	*F*_(2,1982)_ = 1.78	*F*_(2,1862)_ = 3.4^*^	*F*_(2,2001)_ = 6.2^**^
High	974	5.66 [5.56–5.77]	6.95 [6.69–7.20]	11.77 [11.45–12.09]	7.81 [7.70–7.92]	5.39 [5.21–5.57]	14.58 [14.48–14.68]	8.61 [8.52–8.70]	7.44 [7.30–7.57]	8.20 [8.10–8.31]	5.78 [5.60–5.96]	4.61 [4.57–4.66]
Low	794	5.60 [5.48–5.73]	7.83 [7.54–8.13]	10.22 [9.83–10.62]	7.48 [7.34–7.62]	5.09 [4.87–5.30]	14.34 [14.22–14.46]	8.38 [8.26–8.50]	7.45 [7.29–7.61]	8.30 [8.17–8.43]	5.78 [5.56–6.00]	4.49 [4.43–4.54]
Inactive	235	5.81 [5.62–6.00]	7.12 [6.57–7.67]	10.54 [9.87–11.21]	7.66 [7.43–7.89]	5.06 [4.66–5.46]	14.39 [14.18–14.61]	8.63 [8.42–8.83]	6.97 [6.67–7.28]	8.05 [7.81–8.28]	5.18 [4.74–5.61]	4.59 [4.49–4.68]
Covid−19 symptoms		*F*_(1,2002)_ = 1.09	*F*_(1,2002)_ = 2 6.2^***^	*F*_(1,1930)_ = 4.28	*F*_(1,1910)_ = 9.8^**^	*F*_(1,1965)_ = 1.09	*F*_(1,2002)_ = 0.27	*F*_(1,1972)_ = 2.57	*F*_(1,1983)_ = 1.12	*F*_(1,1983)_ = 0.39	*F*_(1,1863)_ = 14.9^***^	*F*_(1,2002)_ = 0.03
No	1,719	5.64 [5.56–5.72]	7.10 [6.91–7.30]	11.13 [10.88–11.39]	7.72 [7.64–7.81]	5.26 [5.12–5.41]	14.47 [14.39–14.55]	8.55 [8.48–8.62]	7.37 [7.26–7.47]	8.23 [8.15–8.32]	5.60 [5.46–5.75]	4.56 [4.53–4.60]
Yes	284	5.75 [5.56–5.94]	8.56 [8.04–9.09]	10.40 [9.76–11.05]	7.32 [7.08–7.56]	5.06 [4.71–5.41]	14.42 [14.22–14.61]	8.36 [8.14–8.58]	7.51 [7.26–7.76]	8.16 [7.95–8.37]	6.31 [5.98–6.64]	4.56 [4.47–4.64]

*** *p < 0.001*;

** *p < 0.01*;

* *p < 0.05*.

### Sociocultural Differences in the Compliance

Table 1 shows the mean scores and 95% confidence intervals for the various scales related to the behavioral, psychosocial, and cognitive variables for participants according to their sociocultural, demographic, and health characteristics. Female participants reported a higher level of compliance with the behavioral recommendations than their male counterparts in the Survey 1 (*M* = 6.5 vs. 6.8, *F*_(1,1999)_ = 35.0, *p* < 0.001) and in the Survey 2 (*M* = 5.4 vs. 5.9, *F*_(1,2002)_ = 61.1, *p* < 0.001). Analyses of covariance also reveal significant differences in the compliance with recommendations among participants as a function of their age group. However, the age gradient in the compliance was much less obvious in the first survey than in second survey, where only younger age (≤ 24 years) was associated with lower score of compliance in the former survey (*M* = 6.4 vs. 6.7, *F*_(1,1999)_ = 21.4, *p* < 0.001). Along with the methodological differences across studies, one possible explanation for this finding is the existence of a delayed effect of public communication about the risk factors for COVID-19 on the adoption of preventive behaviors. Finally, analyses of covariance show that neither the socioeconomic status nor the history of COVID-like symptoms were associated with the behavioral recommendations (*p* > 0.05).

### Associations Between Behavioral, Psychosocial, and Cognitive Variables

The correlations between the various scales related to the behavioral, psychosocial, and cognitive factors are shown in Table 2. Most of these variables were strongly intercorrelated. Notably, with the notable exception of anxiety in Survey 2 (*r* = 0.03, *p* > 0.05), the compliance with behavioral recommendations was significantly associated with all the psychosocial/cognitive variables introduced in the analysis. However, the value of several coefficients changed considerably over time. Some correlations substantially increased in strength (those related to perceived behavioral control and subjective norms), while some others decreased across surveys (those related to perceived cause of infection and perceived effectiveness of preventive behaviors). These results indicate that the weight of the main cognitive factors implicated in the behavioral decision-making tended to change during the early phase of the lockdown.

**Table 2:** **(A)** Pearson Correlations between the various Factors (Survey 1, *N* = 2,000); **(B)** Pearson Correlations Between the various Factors (Survey 2, *N* = 2,003).

	**Adoption of preventive behaviors**	**Anxiety**	**Trust in institutions**	**Perceived susceptibility**	**Perceived severity**	**Worry**	**Perceived cause of infection**	**Perceived behavioral control**	**Perceived barriers of PB**	**Perceived effectiveness of PB**	**Subjective norms**
**(A)**											
Adoption of preventive behaviors	–	0.05^*^	0.08^**^	0.08^***^	0.20^***^	0.16^***^	0.22^***^	0.16^***^	−0.08^***^	0.51^***^	0.27^***^
Anxiety		–	−0.17^***^	0.22^***^	0.18^***^	0.41^***^	−0.06^**^	−0.13^***^	0.21^***^	−0.03	−0.05^*^
Trust in institutions			–	−0.02	0.03	−0.03	−0.05^*^	0.14^***^	−0.03	0.09^***^	0.20^***^
Perceived susceptibility				–	0.28^***^	0.29^***^	-0.03	0.07^**^	0.03	0.04	0.07^**^
Perceived severity					–	0.57^***^	0.08^***^	0.34^***^	−0.14^***^	0.20^***^	0.18^***^
Worry						–	0.08^***^	0.21^***^	−0.02	0.13^***^	0.15^***^
Perceived cause of infection							–	0.14^***^	−0.19 ^***^	0.21^***^	0.14^***^
Perceived behavioral control								–	−0.21^***^	0.17^***^	0.27^***^
Perceived barriers of PB									–	−0.12^***^	−0.09^***^
Perceived effectiveness of PB										–	0.29^***^
Subjective norms											–
**(B)**											
Adoption of preventive behaviors	–	0.03	0.07^**^	0.08^**^	0.25^***^	0.22^***^	0.11^***^	0.29^***^	−0.06^**^	0.22^***^	0.36^***^
Anxiety		–	−0.12^***^	0.25^***^	0.24^***^	0.42^***^	−0.01	−0.07^**^	0.14^***^	−0.11^***^	−0.05^*^
Trust in institutions			–	0.02	0.08^***^	0.03	−0.01	0.15^***^	0.10^***^	0.48^***^	0.19^***^
Perceived susceptibility				–	0.35^***^	0.32^***^	−0.03	0.09^***^	0.05^*^	0.05^*^	0.06^**^
Perceived severity					–	0.59^***^	0.06^**^	0.37^***^	0.01	0.23^***^	0.27^***^
Worry						–	0.08^***^	0.24^***^	0.06^**^	0.15^***^	0.21^***^
Perceived cause of infection							–	0.09^***^	−0.09^***^	0.02	0.07^**^
Perceived behavioral control								–	−0.08^***^	0.34^***^	0.33^***^
Perceived barriers of PB									–	0.07	−0.04
Perceived effectiveness of PB										–	0.31^***^
Subjective norms											–

*Prob (> F)*:

* *p < 0.05*;

** *p < 0.01*;

*** *p < 0.001*.

Then, a hierarchical regression analyses was performed to test the predictors of compliance with behavioral recommendations and guidelines during the study period. Following initial bivariate analyses, a series of multivariable model that considered the predictors in the same theoretical class (i.e., sociocultural, psychosocial, and social cognitive) were employed with the number of protective behaviors recommended by the public health authorities as the dependent variable. The regression coefficients (β) and the variance explained for each class of predictors are displayed in Table 3. Sociocultural variables were entered on step 1, psychosocial variables on step 2, and cognitive variables on step 3. The number of protective behaviors recommended by the public health authorities measuring the participants' compliance with the behavioral regulations and guidelines was the dependent variable.

**Table 3:** **(A)** Hierarchical regression of compliance with public health recommendations on sociocultural, psychosocial, and social cognitive variables (survey 1, *N* = 2,000); **(B)** Hierarchical regression of compliance with public health recommendations on sociocultural, psychosocial and social cognitive variables (survey 2, *N* = 2,003).

			***B*** **(step 1)**		***B* (step 2)**	***B* (step 3)**	**Δ*R*^**2**^ for step^**a**^**	**Total^**b**^*R*^**2**^**
**(A)**								
**1**	**Sociocultural and demographic factors**						0.03^***^	
	Gender	Male	(ref.)					
		Female		0.13^***^	0.12^***^	0.05^*^		
	Age group	18–24	(ref.)					
		25–34		0.11^**^	0.12^**^	0.10^**^		
		35–49		0.14^***^	0.15^***^	0.08^*^		
		50–64		0.16^***^	0.17^***^	0.07^*^		
		65 +		0.12^**^	0.14^**^	0.02		
	Socioeconomic status	High	(ref.)					
		Low		0.03	0.04	0.06^*^		
		Inactive		0.00	0.01	−0.01		
	Covid-19 symptoms	No	(ref.)					
		Yes	−0.03		−0.03	−0.03		
**2**	**Psychosocial factors**						0.01^***^	0.04^***^
	Anxiety				0.04	0.04		
	Trust in institutions				0.08^**^	0.03		
	Social support—instrumental	No	(ref.)					
		Yes			0.00	0.01		
	Social support—informational	No	(ref.)					
		Yes			0.03	0.04		
	Social support—emotional	No	(ref.)					
		Yes			0.04	−0.00		
**3**	**Cognitive factors**						0.23^***^	0.27^***^
	Perceived susceptibility					0.05^*^		
	Perceived severity					0.05^*^		
	Worry					0.00		
	Perceived cause of infection					0.13^***^		
	Perceived behavioral control					0.03		
	Perceived barriers of PB					−0.01		
	Perceived effectiveness of PB					0.37^***^		
	Subjective norms					0.13^***^		
**(B)**								
1	**Sociocultural and demographic factors**							0.06^***^
	Gender	Male	(ref.)					
		Female		0.19^***^	0.18^***^	0.09^***^		
	Age group	18–24	(ref.)					
		25–34		0.14^***^	0.14^***^	0.10^**^		
		35–49		0.21^***^	0.22^***^	0.16^***^		
		50–64		0.26^***^	0.27^***^	0.18^***^		
		65 +		0.29^***^	0.29^***^	0.17^***^		
	Socioeconomic status	High	(ref.)					
		Low		−0.01	0.00	0.02		
		Inactive		0.07^**^	0.09^**^	0.07^**^		
	Covid-19 symptoms	No	(ref.)					
		Yes	0.04		0.04^*^	0.04^*^		
**2**	**Psychosocial factors**						0.01^**^	0.07^***^
	Anxiety				0.00	0.00		
	Trust in institutions				0.07^**^	−0.02		
	Social support—instrumental	No	(ref.)					
		Yes			−0.04	−0.02		
	Social support—informational	No	(ref.)					
		Yes			0.02	0.04		
	Social support—emotional	No	(ref.)					
		Yes			0.06^*^	−0.02		
**3**	**Cognitive factors**						0.23^***^	0.30^***^
	Perceived susceptibility					−0.02		
	Perceived severity					0.07^**^		
	Worry					0.04		
	Perceived cause of infection					0.08^***^		
	Perceived behavioral control					0.18^***^		
	Perceived barriers of PB					−0.07^***^		
	Perceived effectiveness of PB					0.05^*^		
	Subjective norms					0.33^***^		

* *p < 0.05*;

** *p < 0.01*;

*** *p < 0.001*.

a *ΔR^2^ for step (Likelihood-ratio test), step 1 vs. 2: LR chi^2^(5) = 22.64, p < 0.0005; step 2 vs. 3: LR chi^2^(8) = 536.31, p < 0.0001*.

b *Total R^2^, step 1: F_(8,1991)_ = 7.78, p < 0.0001; step 2: F_(13,1986)_ = 6.57, p < 0.0001; step3: F_(21,1978)_ = 34.26, p < 0.0001*.

c *ΔR^2^ for step (Likelihood-ratio test), step 1 vs. 2: LR chi^2^(5) = 20.06, p < 0.002; step 2 vs. 3: LR chi2(8) = 578.07, p < 0.0001*.

d *Total R^2^, step 1: F_(8,1994)_ = 16.17, p < 0.0001; step 2: F_(13,1989)_ = 11.57, p < 0.0001; step3: F_(21,1981)_ = 41.08, p < 0.0001*.

In survey 1, sociocultural variables accounted for 3% of the variance on the step 1 of the regression (*R*^2^ = 0.03, *F*_(8,1991)_ = 7.78, *p* < 0.0001), although only sex and age were significant predictors of compliance with behavioral recommendations. Thus, younger and male participants were less likely to report engagement in preventive behaviors. Age and sex remained significant predictors after psychosocial factors were entered on step 2 (*R*^2^ change = 0.01, LR *chi*^2^(5) = 22.64, *p* < 0.0005), along with trust in institutions (β = 0.08, *p* < 0.01). Social cognitive variables were entered on step 3 and produced a significant increasing in the variance explained (*R*^2^ change = 0.23, LR *chi*^2^(8) = 536.31, *p* < 0.0001). Given the significant reduction in the regression coefficients associated with age and gender, this suggests that the social cognitive variables mediated the effect of on compliance with behavioral recommendations. The final model accounted for 27% of variance (*R*^2^ = 0.27, *F*_(21,1978)_ = 34.26, *p* < 0.0001), with perceived causes of infection, perceived effectiveness of preventive behaviors, and subjective norms as most significant predictors (β = 0.13, 0.37, and 0.13, respectively, all *p* < 0.001).

In survey 2, sociocultural variables accounted for 6% of the variance on the step 1 of the regression (*R*^2^ = 0.06, *F*_(8,1994)_ = 16.17, *p* < 0.0001). Both sex and age were significant predictors of compliance with behavioral recommendations, along with an inactive SES. Interestingly, the older the participants, the more likely they were to report engagement in preventive behaviors recommended by the public health authorities. All the sociocultural variables remained predictive of compliance after the psychosocial variables were entered into the model. Congruent with survey 1, inclusion of these variables only added 1% to the explained variance (*R*^2^ change = 0.01, LR *chi*^2^(5) = 20.06, *p* < 0.002). Trust in institutions was independently predictive of the level of compliance among participants (β = 0.07, *p* < 0.01), such that those who trust the authorities were a bit more likely to report the engagement in preventive behaviors, and emotional support was also marginally predictive (β = 0.06, *p* < 0.05). Cognitive variables were entered on step 3, and again substantially increased the explained variance to a total of 30% (*R*^2^ = 0.30, *F*_(21,1981)_ = 41.08, *p* < 0.0001). In order of magnitude, subjective norms, perceived behavioral control, perceived cause of infection, and perceived barriers of preventive behaviors were independently predictive of compliance (β = 0.33, 0.18, 0.08, and 0.07, respectively, all *p* < 0.001). However, it should be noted that the findings of the survey 2 were less convincing with respect to the hypothesis that social cognitive variables would mediate the effect of sociocultural variables on the reported engagement in preventive behaviors. Indeed, controlling for both psychosocial and cognitive variables, sex and age still exerted a highly significant influence on compliance with behavioral recommendations.

### Mediation Analyses

To test the hypothesis that social cognitive variables mediate the observed effect of sex and age on the compliance with the behavioral regulations and guidelines, we used a SEM program assessing the indirect effects in multiple mediators models. The results of these analyses (*SE*s, critical ratios and *p*-value) are reported in Table 4. In survey 1, by order of magnitude, perceived effectiveness of preventive behaviors, subjective norms, perceived severity, and perceived causes of infection significantly mediated the relation between the participants' sex and engagement in preventive behaviors (*Z* = 4.61, 3.84, 3.04, and 2.92, respectively, all *p* < 0.01). Altogether, these four social cognitive variables accounted for 53% of the total effect of sex on compliance. It was also found that, by order of magnitude, perceived causes of infection, subjective norms, perceived susceptibility, and perceived effectiveness of preventive behaviors significantly mediated the effect on age on compliance with behavioral recommendations (*Z* = 3.16, 2.65, 2.75, 2.30, and 2.50, respectively, all *p* < 0.01). These five social cognitive variables accounted for 51% of the total effect of age on compliance.

**Table 4:** **(A)** Mediation analysis with SEM (structural equation modeling) examining the indirect effects of gender and age group on adoption of prevention behaviors (PB) through cognitive factors (study 1, *N* = 2,000); **(B)** Mediation analysis with SEM (structural equation modeling) examining the indirect effects of gender and age group on adoption of prevention behaviors (PB) through cognitive factors (study 2, *N* = 2,003).

		**Indirect effect**													
**IV**	**M**	**Coeff**.	**S.E**.	***Z***	***p*-value**	**CI (95%) inf sup**		**% of total effect**							
**(A)**															
Gender	Perceived effectiveness of PB	0.061	0.013	4.61	0.000	0.035	0.087	28.4%							
Gender	Subjective norms	0.022	0.006	3.84	0.000	0.011	0.034	10.3%							
Gender	Perceived cause of infection	0.015	0.005	2.92	0.004	0.005	0.024	6.8%						CI (95%)	
Gender	Perceived susceptibility	*0.002*	*0.002*	*0.96*	*0.335*	*−0.002*	*0.005*	*0.7%*		Coeff.	S.E.	*Z*	*p*-value	inf	sup
Gender	Perceived severity	0.015	0.005	3.04	0.002	0.005	0.025	7.0%	Direct effect	0.101	0.031	3.23	0.001	0.040	0.162
	**Total indirect effect**	**0.115**	**0.016**	**7.34**	**0.000**	**0.084**	**0.145**	**53.2%**	Total effect	0.216	0.034	6.33	0.000	0.149	0.282
Age group	Perceived effectiveness of PB	0.223	0.089	2.50	0.012	0.048	0.397	20.4%							
Age group	Subjective norms	0.098	0.037	2.65	0.008	0.025	0.170	8.9%							
Age group	Perceived cause of infection	0.108	0.034	3.16	0.002	0.041	0.175	9.9%						CI (95%)	
Age group	Perceived susceptibility	0.057	0.025	2.30	0.021	0.008	0.106	5.2%		Coeff.	S.E.	*Z*	*p*-value	inf	sup
Age group	Perceived severity	0.074	0.027	2.75	0.006	0.021	0.126	6.8%	Direct effect	0.533	0.209	2.55	0.011	0.124	0.942
	**Total indirect effect**	**0.559**	**0.106**	**5.26**	**0.000**	**0.351**	**0.768**	**51.2%**	Total effect	1.092	0.230	4.75	0.000	0.642	1.543
**(B)**															
Gender	Subjective norms	0.141	0.026	5.40	0.000	0.090	0.192	24.0%							
Gender	Perceived behavioral control	0.070	0.016	4.51	0.000	0.040	0.101	11.9%							
Gender	Perceived cause of infection	*0.007*	*0.006*	*1.14*	*0.254*	*−0.005*	*0.018*	*1.1%*							
Gender	Perceived barriers of PB	0.038	0.011	3.43	0.001	0.016	0.059	6.4%						CI (95%)	
Gender	Perceived effectiveness of PB	*0.011*	*0.006*	*1.74*	*0.082*	*−0.001*	*0.024*	*1.9%*		Coeff.	S.E.	*Z*	*p*-value	inf	sup
Gender	Perceived severity	0.030	0.010	3.07	0.002	0.011	0.049	5.1%	Direct effect	0.291	0.064	4.56	0.000	0.166	0.415
	**Total indirect effect**	**0.296**	**0.033**	**8.98**	**0.000**	**0.231**	**0.361**	**50.5%**	Total effect	0.587	0.069	8.48	0.000	0.451	0.722
Age group	Subjective norms	0.540	0.180	2.99	0.003	0.186	0.894	24.7%							
Age group	Perceived behavioral control	*-0.027*	*0.097*	*−0.280*	*0.778*	*−0.218*	*0.163*	*−1.3%*							
Age group	Perceived cause of infection	*−0.045*	*0.043*	*−1.06*	*0.290*	*−0.129*	*0.039*	*−2.1%*							
Age group	Perceived barriers of PB	*0.059*	*0.048*	*1.23*	*0.219*	*−0.035*	*0.152*	*2.7%*						CI (95%)	
Age group	Perceived effectiveness of PB	*0.043*	*0.031*	*1.42*	*0.157*	*−0.017*	*0.103*	*2,0%*		Coeff.	S.E.	*Z*	*p*-value	inf	sup
Age group	Perceived severity	0.256	0.074	3.44	0.001	0.110	0.401	11.7%	Direct effect	1.365	0.450	3.04	0.002	0.484	2.249
	**Total indirect effect**	**0.825**	**0.228**	**3.62**	**0.000**	**0.378**	**1.272**	**37.7%**	Total effect	2.190	0.496	4.41	0.000	1.217	3.162

*IV, independent variable; M, potential mediator; DV, dependent variable (number of preventive behaviors adopted)*.

In survey 2, subjective norms, perceived behavioral control, perceived barriers to preventive behaviors, and perceived severity of COVID-19 were found to significantly mediate the effect of sex on the behavioral response of participants (*Z* = 5.40, 4.51, 3.43, and 3.07, respectively, all *p* < 0.01). These four social cognitive variables accounted for 47% of the total effect of age on compliance. By contrast, only subjective norms and perceived severity of the disease significantly mediated the relation between and engagement in preventive behaviors (*Z* = 2.99, and 3.44, respectively, all *p* < 0.01). These two social cognitive variables accounted for 36% of the total effect of age on compliance with behavioral recommendations. Overall, these results confirm that the effect of gender and age on the adoption of preventive behaviors recommended by the public health authorities is mediated to a large extent by a few social cognitive variables(from 38 to 53%), including systematically to the social norms perceived by the participants.

## Discussion

In the absence of effective pharmaceutical interventions, such as vaccines or antiviral medicines, the COVID-19 pandemic has required rapid and massive changes in individual and social behaviors in order to control and prevent the spread of the disease around the world. Over the course of the last few months, most epidemiologists and public health experts argued that large-scale adoption of health protective measures related to hygiene and physical distancing was a crucial strategy for reducing the rate of morbidity and mortality from COVID-19. Nevertheless, the promotion of social isolation for the sake of health protection interferes with the fundamental human need to connect, communicate, and interact with others, which is generally associated with better mental and physical health status (Cowling et al., 2020). Therefore, this unexpected and unique situation in the modern age of public health systems has posed a considerable economic, psychological, and behavioral burden on individuals and communities in both developed and developing countries. Noticeably, the pandemic has led some experts to consider that refusal or hesitancy to comply with the behavioral recommendations and guidelines of governments and public health authorities may represent a major “threat to national health and security” (Mansdorf, 2020). Thus, in many countries like France, it has been assumed by policy-makers and medical experts that the required shifts in behavioral patterns within the population could not be solely achieved through the development of education and awareness campaigns promoting a range of preventive behaviors. This assumption caused the French government to declare the state of national emergency, which permits the implementation of significant behavioral change by law reinforcement. However, for the nations who particularly value individual freedom and display weaker social norms and conventions, the compliance with a range of preventive measures which seriously restricts the protection of fundamental civil liberties remains challenging (Van Bavel et al., 2020). Overall, our data did not support the assumption that non-compliance with recommendations and regulations represented a genuine threat to public health in France. On the contrary, a very large majority of participants reported in both surveys that they have adopted the health protective behaviors which were recommended in the guidelines provided by the public health authorities, regardless of their coercive or non-coercive nature. Overall, these results question the pessimistic view on the capacity of people from “permissive” societies to adapt significantly their social behaviors and norms in the face of a serious emerging health threat. In line with our findings, a vast international study of approximately 8,000 individuals across 70 nations, conducted after the lockdown orders, showed that French respondents were significantly more likely to report changes in their behavior and compliance with the guidelines provided by the public health authorities than English or German respondents (Clark et al., 2020). This might be very surprising for many observers as French culture is often depicted as a very individualistic one, with lower commitment of the citizens in social norms and higher tolerance for “deviance,” especially when compared to some other European cultures (Germany, Norway, Portugal).

Unsurprisingly, there were however significant individual differences in the degree of compliance with these recommendations that requires a better understanding in order to design and develop relevant behavioral interventions. As noted by Van Bavel et al. (2020: 460), “the social and behavioral sciences can provide valuable insights for managing the pandemic and its impacts.” The purpose of our research was to examine the role of a variety of sociocultural, psychosocial, and cognitive factors in the adoption of the preventive behavior recommended or imposed by the public health authorities. On the one hand, the results confirm some expected sociocultural and demographic variations in the French population as men and younger adults were less likely to follow the guidelines aiming to contain the spread of COVID-19. On the other hand, among the psychosocial variables included in the analyses, only the trust in government was found to be associated with the self-reported engagement in preventive behaviors promoted by the authorities across surveys. Even though the adoption of preventive behaviors was significantly higher among participants who were more anxious and had more trust in government, this did not seem to explain directly their higher level of compliance with recommendations. As shown by the hierarchical regression analysis, there were either much less (survey 2) or no longer (survey 1) significant differences in compliance with behavioral recommendations across the subgroups of participants after incorporating these factors in the overall model, which give strong empirical support the assumption that differences in beliefs and expectations are a fundamental pathway to social differences in preventive behaviors. Further mediation analyses using SEM programs shows that social cognitive variables mediate about 50% of the effect of sex on the compliance with the behavioral recommendations, while they mediate from to 38 to 51% of effect of age on the same variable, in Survey 2 and Survey 1, respectively.

As pointed out earlier, only the incorporation of the sociocognitive variables in the regression analysis was actually found to considerably improve the explanatory power of the model (with a *R*^2^ change = 23% in both surveys). This result indicates that sociocognitive factors might play a substantially more important role than sociocultural and psychosocial factors in the adoption of preventive health behaviors. That does not necessarily mean that more distal factors derived from more general social psychological theories, such as trust or anxiety, should be neglected as they might have more stable influence on preventive behaviors over time (Plohl and Musil, 2020; Roozenbeek et al., 2020). Nevertheless, this makes it crucial to determine what are the most influential socio-cognitive factors implicated in the behavioral decision-making in response to the COVID-19 pandemic. Noticeably, the data collected in the research show a significant shift over time since the perceived effectiveness of the preventive behaviors—and to a lesser extent the subjective norms—were by far the most influential determinants of compliance in the first survey, while the subjective norms represented in the overall model the most important predictor in the second survey. This finding suggests that the perceived behavioral norms play a growing role in the compliance with the recommendations by the health authorities during the early phase of the lockdown, and therefore should be one of the main targets of public health interventions aiming to promote risk reduction measures. Although this factor has been largely neglected in the previous research devoted to the H1N1 pandemic (Bish and Michie, 2010; Brien et al., 2012), this result is not a surprise as many other researchers have suggested that health preventive behavior is strongly influenced by social norms (Reid et al., 2010; Sheeran et al., 2016; Raude et al., 2019).

### Limitations

This study may be prone to a number of methodological limitations, which are common in questionnaire survey, such as the discrepancy between actual and self-reported health behaviors which are caused by the social desirability bias in response to some questions (King and Bruner, 2000). However, it should be noted that responses to online and self-administered questionnaire seem to be less biased than to face-to-face or telephone interviews. For instance, Weinstein and his colleagues found in a review of literature on smoker's risk perceptions that respondents were more likely to reveal their unrealistic optimism in self-administrated questionnaire than in face-to-face interviews (Weinstein et al., 2005). Furthermore, the unexpected high rate of compliance observed through our cross-sectional surveys within the French population can be externally validated by other types of data documenting the dramatic change in social and individual behaviors, such as those related to mobility or road traffic. According to a recent report by the National Institute of Road Safety, the France's lockdown order on March 17 has had the unprecedented effect of dividing the number of traffic accidents by four, as well as crash-related injuries and fatalities by more than two, when compared to the previous years (Les Décodeurs, 2020). In the same vein, the mobility data collected by Google in France among Android mobile users show a sharp drop in attendance at grocery and pharmacy (−72%), parks (−82%), and transit stations (−87%) 2 weeks after the implementation of the lockdown (GOOGLE, 2020). By and large, this objective data confirm the high rate of adoption of public health recommendations and regulations revealed by our surveys.

Another methodological limitation derived from the utilization of single items in our surveys to represent some key concepts related to the social cognitive approach, such as the perceived susceptibility or the perceived severity of COVID-19. As underlined by McIver and Carmines (1981), the biggest problem with single item measures is that one cannot estimate their measurement properties, including their reliability and validity. Moreover, it should be noted that self-reported engagement in preventive behaviors recommended by the public authorities was used in our studies as an indirect measure of the “compliance” within the French population. Hence some divergences may have occurred between the concept and its measurement as taking a specific preventive actions does not necessarily imply that participants wanted to comply with the health recommendations. Finally, the three classes of explanatory variables addressed in the analysis included a limited range of potential predictors, so that some other important social and psychological factors related to health behaviors might have been neglected in the analysis. This is because we constrained ourselves to limit the number of items included in the questionnaire in order to ensure high completion rates, as well as the quality of the collected data.

## Conclusion

To conclude, congruent with the results of international studies conducted during the same period (Clark et al., 2020), our study show that the French population exhibited a high rate of compliance with the public health recommendations and guidelines, which questions the pessimistic view on the capacity of French people to adapt significantly and quickly their social norms in the face of a serious health threat. Spite of a high rate of compliance in the whole population, some expected differences were observed among the subgroups in terms of behavioral response to the COVID-19, with the men and younger participants being less likely to comply with the recommendations. However, when the sociocognitive variables were entered into the overall model, in particular the subjective norms, the regression coefficients for both the sociocultural and psychosocial factors were either substantially lower or no longer significant, demonstrating that these latter variables were more indirect than direct predictors of compliance with recommendations. This suggests that people's behaviors associated with COVID-19 might be amenable to improvement. Rather than appealing to fear of punishment or harm, we would encourage policy-makers and public health experts based on the data presented here to emphasize on positive norms in their messaging used to promote adaptive health behavior, as it cannot be overlooked that their perceptions remains inaccurate within large segments of the most vulnerable populations. Finally yet importantly, future systematic research of the interaction between sociocultural and social cognitive variables is required to better understand potential relapse in a variety of preventive behaviors over the COVID-19 pandemic. More specifically, ideological orientations and worldviews might be useful predictors of compliance with health recommendations (Ward et al., 2020).

## Data Availability Statement

The raw data supporting the conclusions of this article will be made available by the authors, without undue reservation.

## Ethics Statement

The studies involving human participants were reviewed and approved by Ethics committee of the University Hospital Institute (IHU) “Mediterranee Infection” (Marseille, France). The ethics committee waived the requirement of written informed consent for participation.

## Author Contributions

JR wrote the article. JR, J-ML, LL, CL, RG, EdR, and PA designed the study, analyzed the data, discussed the results, and reviewed and approved the article. All authors contributed to the article and approved the submitted version.

## Conflict of Interest

The authors declare that the research was conducted in the absence of any commercial or financial relationships that could be construed as a potential conflict of interest.
